# Electrically controlled white laser emission through liquid crystal/polymer multiphases

**DOI:** 10.1038/s41377-020-0252-9

**Published:** 2020-02-11

**Authors:** Alina Adamow, Adam Szukalski, Lech Sznitko, Luana Persano, Dario Pisignano, Andrea Camposeo, Jaroslaw Mysliwiec

**Affiliations:** 1 Faculty of Chemistry, Wroclaw University of Science and Technology, Wybrzeze Wyspianskiego 27, 50-370 Wroclaw, Poland; 2 NEST, Istituto Nanoscienze-CNR and Scuola Normale Superiore, Piazza S. Silvestro 12, 56127 Pisa, Italy; 3 Dipartimento di Fisica, Università di Pisa, Largo B. Pontecorvo 3, 56127 Pisa, Italy

**Keywords:** Solid-state lasers, Liquid crystals, Polymers

## Abstract

White lasers are becoming increasingly relevant in various fields since they exhibit unprecedented properties in terms of beam brightness and intensity modulation. Here we introduce a white laser based on a polymer matrix encompassing liquid crystals and multiple organic chromophores in a multifunctional phase-separation system. The separation of the hydrophilic matrix and the hydrophobic liquid crystals leads to the formation of a complex optically active layer, featuring lasing emission tuneable from blue to red. White laser emission is found with an optical excitation threshold of approximately 12 mJ/cm^2^. Importantly, an external electric field can be used to control the device emission intensity. White lasers with low-voltage (≤10 V) controllable emission might pave the way for a new generation of broadband light sources for analytical, computational, and communication applications.

## Organic lasers: Polymer suspensions see the white

A laser that can emit white light has been fabricated by mixing liquid crystal particles and polymers into an emulsion. Devices that use organic compounds to produce white light are normally fabricated by combining differently coloured lasers together. Camposeo Andrea from Italy’s Istituto Nanoscienze-CNR and colleagues have now achieved similar effects in a more compact set-up by utilizing phase separations between water-repelling and water-attracting molecules. The team incorporated hydrophobic liquid crystal droplets, which emit green or red light, into a hydrophilic alcohol containing blue-emitting compounds. Analysis of the polymer emulsion revealed that it had a layered structure that may enhance both individual colour emissions and the blending into white light. Sandwiching the organic laser between a pair of transparent electrodes enabled the authors to manipulate the white laser intensity with a small voltage.

## Introduction

Controllable systems for generating intense, multicolour, and white light are introducing new paradigms in many applications, including spectroscopy^1^, optical sensing^2^, communication^3^, lighting^4^, and display technologies^5^. The high directionality, brightness, and capability to modulate the intensity of multicolour light sources are highly important in this respect, allowing for enhancement of the signal-to-noise ratio in spectroscopic systems, the sensitivity in detection devices, data transmission rates, and the colour rendering in lighting elements. This has motivated researchers to develop multicolour and white laser sources, which inherently feature the aforementioned properties. Lasers are routinely realized with emission at a well-defined, single wavelength by using various materials and cavity geometries. Multicolour and white lasers that encompass active media with broadband optical gain are generally much more challenging. For instance, a monolithic white laser has recently been realized by using multisegment heterostructure nanosheets made of ZnCdSSe alloys^6^.

In the framework of broadband laser devices, multicolour or white light-emitting systems that exploit organic compounds in their active layers are especially interesting since they can be easily tuned and embedded in portable and wearable platforms for diagnostics, communication, and anti-counterfeiting^7–12^. Spectral tunability and large stimulated emission cross-sections can be realized in organics either by chemical synthesis and functionalization or by blending various fluorescent molecules in dielectric and transparent polymer matrices^11,12^. Recent advances have allowed pulsed and quasi-continuous-wave coherent emission to be obtained at single frequencies under incoherent ultraviolet excitation by light-emitting diodes^13,14^, as well as pumping in the near-infrared via two-photon and multiphoton excitation^15^. Many cavity architectures have been explored for organic lasers in the visible and near-infrared spectral range, showing low excitation thresholds, narrow linewidths, and broad spectral tunability^11,12^. Demonstrated architectures include various disordered and random structures^16^ utilizing complex inhomogeneous materials such as nanofibres^17,18^, organic crystals^19^, biomaterials^20^, and bio-mimicking and bio-derived materials^21,22^. Different from conventional lasers where an external cavity determines the optical modes, in such systems, the conditions for lasing are affected by multiple scattering in amorphous materials^16,23^. Light is amplified along its random walk in disordered media that exhibit optical gain, leading to either intensity feedback random lasing or resonant feedback random lasing^24,25^. The former is characterized by a broad and smooth lasing spectrum, with light amplified mainly by diffusional processes. In contrast, in resonant feedback random lasing, relevant interference effects can occur, and emission typically features multiple narrow peaks. Random architectures can potentially lase in a broad spectral range by combining them with a disordered sample morphology, which provides multiple scattering, with organic materials featuring stimulated emission over large intervals of wavelength.

However, laser systems that emit white light and are based on organic active materials have only been reported in a very few pioneering works^26–29^. Such organic white lasers (oWLs) are mostly achieved by combining the output beams of individual lasers, each emitting at a single (blue, green, and red) colour. Unfortunately, the emission of such lasers cannot be modulated by external electrical or optical signals. In this respect, multiphase optical media, such as emulsions^30^, might provide desirable features when loading them with chromophores with varied optical gain bands, as well as with compounds responsive to external stimuli for emission modulation (spectrum, polarization, or intensity). In addition, such heterogeneous compositions, with associated spatial variations in the refractive index and optical gain, efficiently function to induce coherent light emission by random lasing.

Here we report multiphase devices supporting broadband and white laser emission. The active material is composed of a hydrophilic, solid polyvinyl alcohol (PVA) matrix, and the phase-separated hydrophobic liquid crystalline mixture E7 (Merck). The incorporation of liquid crystals (LCs) into optical media is very effective for controlling the polarization, wavelength, intensity, and scattering properties of organic lasers^31,32^. LCs show long-range orientational order, optical anisotropy, and fluid-like behaviour. Moreover, their molecular alignment can be easily changed by controlling the temperature and electric or magnetic fields^33^ and via proper molecular and surface interactions^30^. For multicolour light generation, a blue-emitting molecule is incorporated into PVA, whereas the LC droplets contain either a green- or a red-emitting component. The resulting multiphase disordered material undergoes random lasing at various colours, including white light at an excitation fluence of 12 mJ/cm^2^. The intensity of the oWL devices can be varied by applying an external direct current (DC) voltage in the range of 0–10 V without degrading the white light emission. Based on these results, notably, the electrically controllable intensity, multiphase oWLs constitute very promising components for diagnostic, lighting, and communication platforms.

## Results

**Fig. 1:**
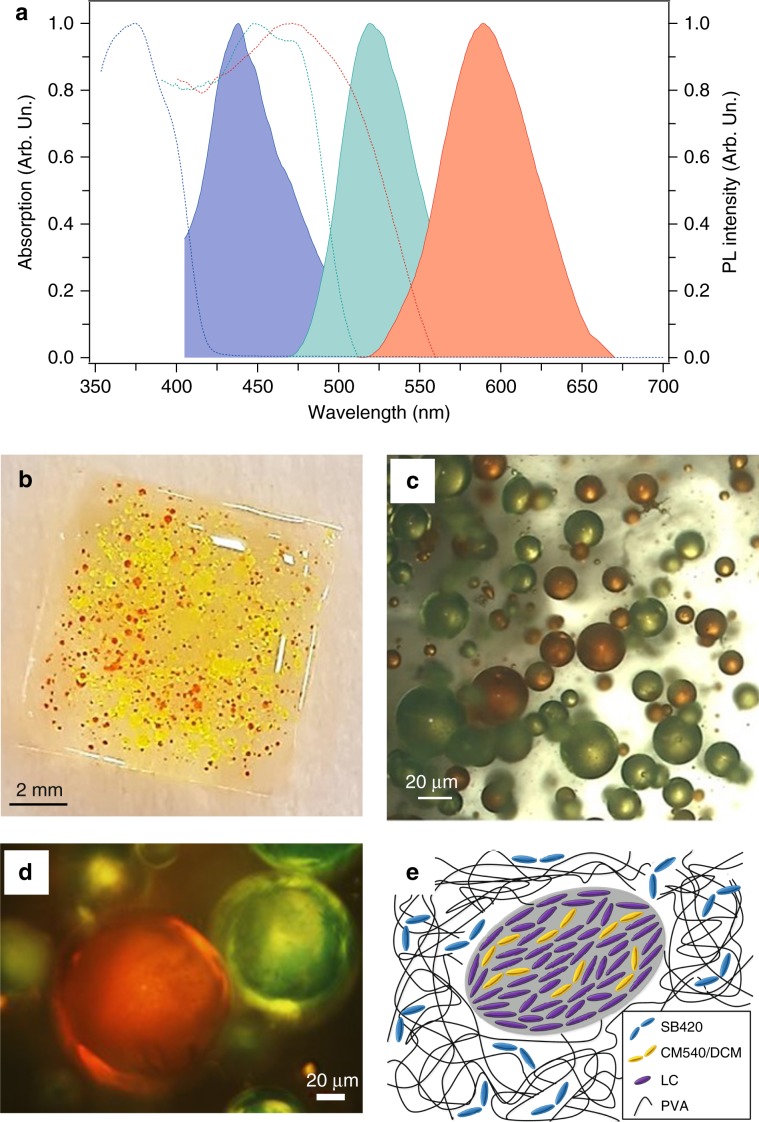
Optical properties and morphology of multiphase active systems. **a** Absorption (dashed lines) and photoluminescence (continuous lines) spectra of the three elementary light-emitting materials. Blue lines: PVA/SB420; green lines: PVA+LC/CM540; red lines: PVA+LC/DCM. **b** Sample photograph: SB420 and LC droplets are embedded in the PVA matrix. CM540 and DCM molecules are incorporated into the LC droplets. **c**, **d** Corresponding optical bright field micrograph (**c**) and optical micrograph acquired with crossed polarizers (**d**). CM540- and DCM-containing droplets appear green and red, respectively. **e** Graphical representation of a cross-section of a region surrounding a single LC droplet in the multiphase system. SB420 molecules are uniformly distributed in the PVA matrix, and the LC molecules form phase-separated droplets surrounded by PVA

Devices are prepared following the procedure schematized in Fig. S1. Emulsions are obtained by sonication of LC/chromophore mixtures and PVA dissolved in deionized water. The choice of PVA is advantageous for preventing flocculation of the LC droplets through steric stabilization^30^. The process involves three elementary optically active materials that can be combined in various forms: (i) PVA with blue-emitting 2,2’-([1,1’-biphenyl]-4.4’-diyldi-2,1-ethenediyl)bis-benzenesulfonic acid disodium salt (SB420), (ii) PVA with LC droplets containing green-emitting (3-(2-benzothiazolyl)-7-(diethylamino)-2H-1-benzopyran-2-one) (CM540), and (iii) PVA with LC droplets containing red-emitting 4-(dicyanomethylene)-2-methyl-6-(4-dimethylaminostyryl)-4H-pyran (DCM), with emission peaks at 438, 519, and 590 nm, respectively (Fig. 1a).

The laser device embedding the multiphase active system is realized in a layer-by-layer fashion by spin-casting a film of PVA/SB420 on top of a quartz substrate, followed by films of PVA/LC/CM540 and PVA/LC/DCM (details in “Materials and methods”). This approach minimizes coalescence and mixing of the phase-separated LC/dye droplets, while diffusion of SB420 in PVA provides a uniformly distributed blue component around the green and red droplets. Other tested methods, such as directly adding LC/CM540 and LC/DCM mixtures to the PVA/SB420 solution followed by single layer casting, lead instead to coalescence of CM540- and DCM-incorporating droplets, i.e., to poor control of the ultimately achieved composition.

A photograph of the resulting sample is displayed in Fig. 1b. The average distance between adjacent droplets in PVA is approximately 170 μm, and the droplet size ranges between 10 and 300 μm (average size ∼100 μm, Fig. S2), as highlighted by optical microscopy (Fig. 1c). In Fig. 1c, one notes that some of the droplets are blurred, being positioned out of the focal plane as a consequence of the three-dimensional (3D) architecture of the sample. The presence of LC ordering in the droplets is confirmed by the polarized optical microscopic (POM) image, showing bright green and red droplets immersed in a dark background (Fig. 1d). Here incident polarized light can only be detected through the analyser with an axis at 90° with respect to that of the polarizer in the presence of an optically anisotropic medium, such as a birefringent sample, which causes rotation of the polarization of the incident light and a consequent non-zero transmission through the analyser. POM analysis allows us to clearly identify the phase separation between the birefringent LC domains and the isotropic polymer matrix, appearing as a dark uniform background. Therefore, two features can be inferred from Fig. 1d, namely, (i) the presence of LC in the droplets and (ii) their planar alignment as a characteristic of the nematic order, as sketched in Fig. 1e. Different from previous evidence of cholesteric order of LCs inside individual microdroplets, with an onion-like arrangement forming a well-ordered photonic structure^30^, here the LC molecules lie in a planar configuration. An analogous, conformal alignment is expected for the CM540 and DCM dopants^34–36^.

**Fig. 2:**
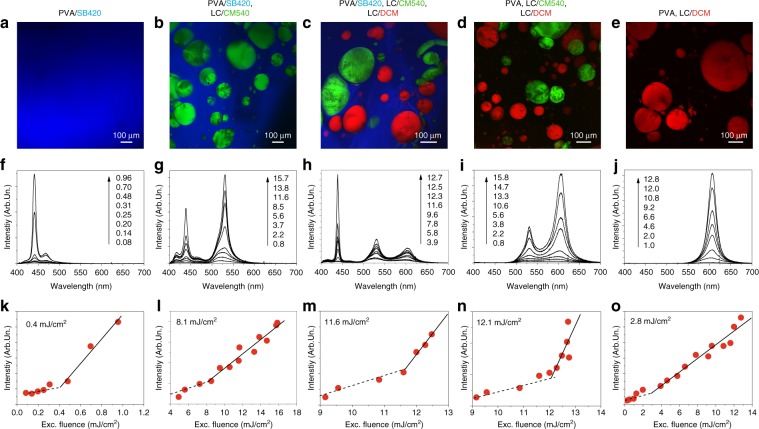
Confocal fluorescence and lasing characteristics of oWLs. **a**–**e** Confocal fluorescence micrographs of samples containing different combinations of blue-, green-, and red-emitting components: **a** PVA with SB420, **b** PVA with SB420 and LC/CM540, **c** PVA with SB420, LC/CM540, and LC/DCM, **d** PVA with LC/CM540 and LC/DCM, and **e** PVA with LC/DCM. **f**–**j** Corresponding lasing spectra obtained with different excitation fluences of the samples shown in (**a**–**e**), respectively. Excitation fluence values (in mJ/cm^2^) are shown in each panel and correspond to the shown spectra (from bottom to top). **k**–**o** Corresponding plots of the emitted intensity vs. the excitation fluence. The dashed and continuous lines are linear fits to the data in the range of excitation below and above the threshold, respectively. Measured threshold values are given at the top of each plot

The emission spectral features and the resulting colour of the multiphase system can be tailored by simply varying the combination of the elementary optically active materials. Figures 2 and S3 summarize the emission properties of a series of samples with individual blue, green, and red colours and their binary and ternary combinations. Fluorescence confocal microscopy supports the occurrence of phase separation between PVA and the LC droplets (Figs. 2a–e and S3a, b) and allows the dyes incorporated in each region to be easily identified. For instance, samples functionalized with SB420 appear as uniform blue-emitting layers (Fig. 2a), whereas those with LC/CM540 and LC/DCM in pristine PVA display green- and red-fluorescent droplets in a dark matrix (Figs. 2e and S3a). These images allow diffusion of CM540 and DCM into PVA, as well as potential mixing with SB420, to be ruled out in the multicomponent system (Fig. 2c). Figures 2f–j and S3c, d show the emission spectra of these materials for various excitation conditions. Clear emission line narrowing is found upon increasing the excitation fluence, with the full width at half maximum (FWHM) decreasing from 62 to 9 nm for SB420 and from 48 and 74 nm to 17 and 24 nm for LC/CM540 and LC/DCM, respectively (Figs. 2f, j, S3c, and S4). Interestingly, the same behaviour is found for each spectral band of the multicomponent systems. Furthermore, all the samples exhibit a well-defined threshold in their light-in light-out (*L*–*L*) plots, in the range of 8–12 mJ/cm^2^ (Figs. 2k, o and S3e).

**Fig. 3:**
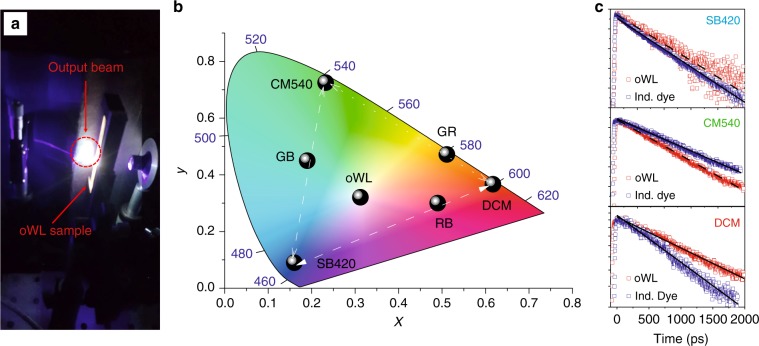
Properties of oWLs devices. **a** Photograph of the oWL device under optical excitation, showing the white light-emitted beam. **b** CIE chromaticity coordinates above the lasing threshold for devices with various combinations of the blue-, green-, and red-emitting components: SB420: PVA with SB420; RB: PVA with SB420 and LC/DCM; DCM: PVA with LC/DCM; GR: PVA with LC/CM540 and LC/DCM; CM540: PVA with LC/CM540; GB: PVA with SB420 and LC/CM540; oWL: PVA with SB420, LC/CM540, and LC/DCM. **c** PL intensity temporal profiles for the three chromophores measured in an oWL sample (red symbols) and in samples with the individual dyes (blue symbols). The vertical axis is in a logarithmic scale. Dashed and continuous lines are exponential fits to the data of oWLs and individual dyes, respectively

The oWL output beam, imaged on a screen, is shown in Fig. 3a. The white emission is bright and directional, with a 6.4° overall measured divergence of the output laser beam (Fig. S5). Overall, the occurrence of white lasing is supported by the decrease in the linewidth (Fig. S6), by the threshold-like behaviour of the *L*–*L* characteristics, and by the well-defined and directional output beam above the excitation threshold. The oWL device shows an operational half-life (*τ*_1/2_, defined as the number of excitation pulses at which the emission intensity drops to half of its initial value) of approximately 5 × 10^4^ excitation pulses under continuous operation in ambient conditions (Fig. S7), in line with other classes of organic lasers^37–39^. The operational lifetime of these oWLs can be further improved by operating them in vacuum conditions (Fig. S7) and, in perspective, by device encapsulation^14,37,40^. Figure 3b summarizes the CIE chromaticity coordinates of the devices analysed in Figs. 2 and S3. The CIE coordinates are calculated for the emission spectra at an excitation fluence of 15 mJ/cm^2^, above the threshold for lasing, and highlight the possibility of precisely calibrating the emission colour and obtaining balanced white light (CIE coordinates of 0.31, 0.32, and 0.37). The white emission can also be finely tuned from cool to warm tones by slightly varying the excitation intensity (Fig. S8). Figure 3c compares the photoluminescence (PL) decay for the three active components in the oWLs with that measured for the individual chromophores. The PL decays exponentially for all the systems, with the lifetimes reported in Table S1. An increase in the fluorescence lifetime of DCM is observed upon embedment in the oWL, together with a slight decrease in the CM540 fluorescence lifetime and a stable SB420 fluorescence lifetime of approximately 0.7–0.8 ns.

**Fig. 4:**
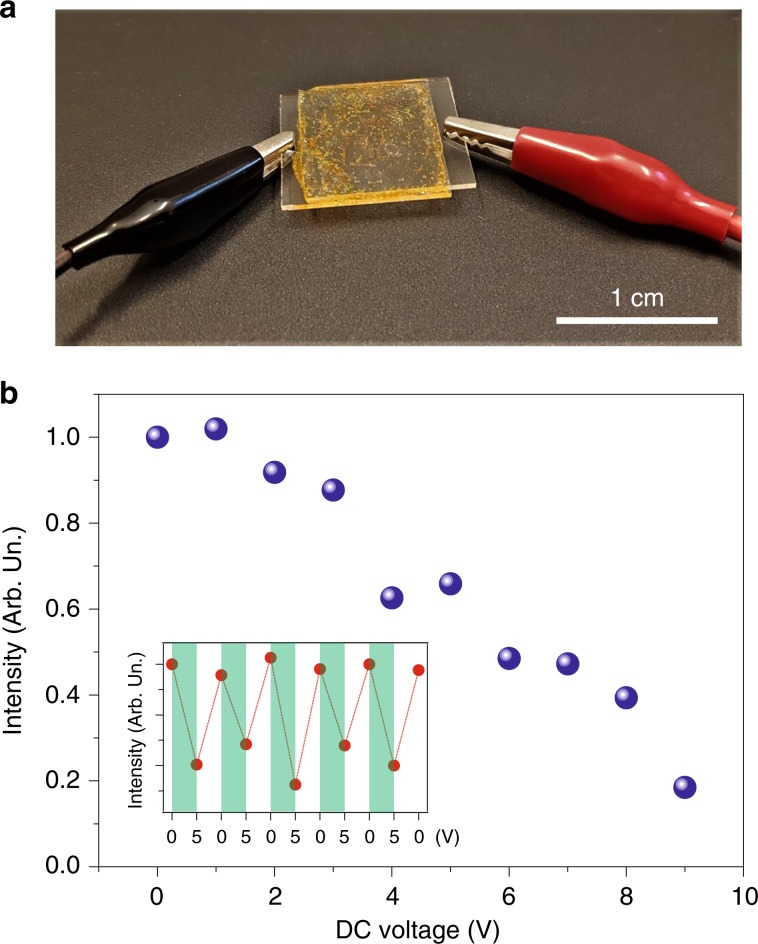
Control of the device emission by external electric field. **a** Photograph of an oWL device whose emission intensity is controlled by an external applied voltage. **b** oWL emission intensity vs. applied DC voltage. The device is pumped at an excitation fluence of 11.9 mJ/cm^2^. Inset: lasing intensity for consecutive voltage cycles of 0–5 V applied to the device

Importantly, the emission of the oWLs can be controlled by an external DC field. This property directly comes from the embedded LC molecules, whose director (i.e., direction of the long-range average orientational order of the LC domains) aligns with the applied electric field. Figure 4a shows a picture of an oWL device with suitable electrical contacts, namely, the active material is sandwiched between two conductive and transparent indium tin oxide (ITO) layers deposited on quartz substrates. In this way, the oWL emission intensity is directed, with a variation up to approximately 80% of the maximum value for applied voltages up to 10 V (Fig. 4b). This allows the oWL output to be easily controlled by switching on and off the applied field (inset of Fig. 4b). In contrast, the spectral emission features of the oWLs are not affected by the electric field, with the CIE coordinates almost unchanged upon repeatedly switching on and off the electric field (Fig. S9). Similarly, the spatial profile and divergence of the output beam are stable upon switching on the electric field, with measured variations <3% (Fig. S10).

## Discussion

Various mechanisms might affect this white lasing, related to the complex and inhomogeneous oWL structure. The above-threshold spectral linewidth (9–24 nm) and the lack of sharp emission peaks are indicative of intensity feedback random lasing due to diffusive amplification of light, as found in various polymers doped with LCs and chromophores^32,41–43^. Indeed, the contrast between the ordinary (*n*_o_ = 1.53) and extraordinary (*n*_e_ = 1.75) refractive indices of the LC domains^32^ and the PVA matrix (*n*_PVA_ = 1.48–1.50 in the visible spectral range^44^) leads to light scattering through the complex material. The characteristic transport length, *l*_T_, for light propagating in the disordered optical medium with LC droplets is expected to be of the same order of magnitude as the droplet average distance, i.e., ≅10^2^ µm, much smaller than the excitation slab length (5 mm, see “Materials and methods”), thus priming a regime of multiple scattering.

Moreover, the slab geometry favours in-plane waveguiding and diffusion of light. Waveguiding enhances the probability of generating stimulated emission, and it might lead to optical interplay of components emitting at different colours. In fact, along the slab, photons emitted by components with emission at higher energies can be absorbed by those with emission at lower energies. This effect would enhance the population inversion of low-energy chromophores, decreasing their threshold for lasing and concomitantly increasing the threshold for high-energy emitters. Indeed, while the single dyes have different excitation thresholds in the range of 0.4–9.1 mJ/cm^2^, the thresholds values for the individual components in the oWL spectra are quite close (≅7–12 mJ/cm^2^, Fig. S6), which is indicative of the interaction triggered by re-absorption and stimulated emission events. Finally, nonradiative energy transfer processes can be sustained at the interface between PVA/SB420 and the LC/dye droplets. Overall, the PL decay data are consistent with the contribution to the decrease of the blue component mainly coming from the bulk PVA/SB420 regions (see Fig. 2c). Indeed, these regions account for by far the largest fraction of SB420 excited within the pumped volume (excitation spot diameter approximately equal to a few hundreds of μm), while nonradiative energy transfer involving SB420 molecules at the interface with droplets is expected to occur to a minor extent within the excitation spot.

**Fig. 5:**
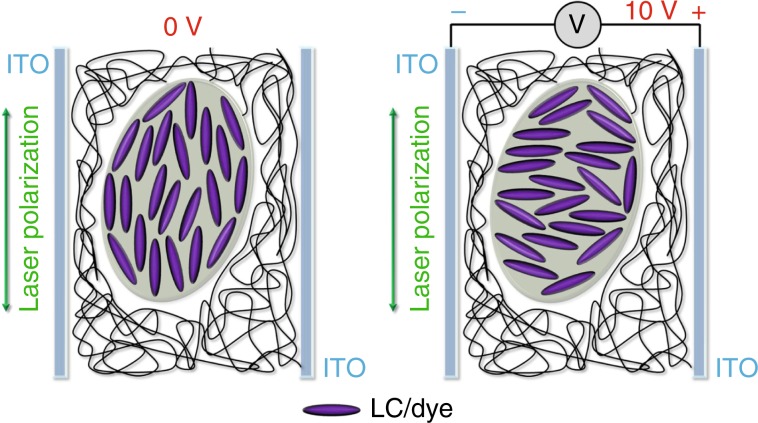
Schematic graphical representation of the effect of a DC electric field on the alignment of LC molecules

Finally, to rationalize the behaviour of the oWLs controlled by an external DC field, one has to consider that the dipole moments of LCs featuring positive dielectric anisotropy, such as the one used here, E7, are oriented parallel to the applied electric field. At zero voltage bias, the orientation of LC molecules is mainly driven by the polarization of the pumping laser, as schematized in Fig. 5, thus enhancing the absorption of the dyes embedded in the LC, which are expected to be aligned parallel to their surrounding LC molecules^34–36^. In contrast, upon activating the voltage bias between the device electrodes, the LC molecules and incorporated chromophores align along the new field direction, namely, perpendicular to the polarization of the excitation light. This effectively decreases the number of absorbed excitation photons and, consequently, the intensity of the green and red components of the oWL emission. Furthermore, a transition from a 3D, isotropic diffusive regime to a two-dimensional, anisotropic multiple scattering regime for light within the slab waveguides can occur upon switching on an external electric field, which would shift the refractive index seen by light to a higher extraordinary value (*n* = 1.75)^32^. Such variation of the paths accessible to photons might especially affect the blue component of the oWL emission, which would be sensitive to the multiple scattering induced by the LC droplets and to absorption by the dyes embedded in the LC. Hence, the intensity of the blue component would also be decreased, thus balancing the overall spectral emission of the oWL. We anticipate that LCs with negative dielectric anisotropy, which align perpendicular to external fields, can instead be exploited to enhance the intensity of the oWL upon applying a voltage bias^45^. Overall, the multiphase optical media developed here can act as very versatile lasing systems whose stimuli-responsive gain material can provide not only spectral tunability and white light emission but also tight emission control using low-voltage DC signals.

In conclusion, organic laser devices with white light emission based on multiphase optical media containing liquid crystalline emulsions are introduced. The combination of various chromophores in a polymer matrix and/or in LC droplets allows single-colour and multicolour laser emission to be achieved in the visible range through compositional control, with an excitation threshold of approximately 12 mJ/cm^2^. Moreover, modulation of the lasing intensity is implemented by applying a low voltage to the device, directing LC and chromophore alignment. These collective results suggest the utility of multiphase materials and lasers made with them in a variety of communication platforms, optical sensors, and diagnostic devices, with the potential for establishing innovative design rules for oWLs in large areas.

## Materials and methods

### Device realization

The steps of device realization are shown in Fig. S1. PVA powder (Mw = 85–124 kDa, Sigma-Aldrich) is dissolved in deionized water at a concentration of 7.5% weight/weight (w/w). Afterwards, 0.4 mg of SB420 (Exciton Inc.) is dissolved in 1 mL of PVA water solution by mechanical mixing for 1 min. CM540 and DCM (Exciton Inc.) are separately dissolved in a LC (E7, Merck) by utilizing 1.5 mg of each dye and 0.3 mL of E7. PVA/LC emulsions are obtained by mixing 20 µL of each LC/dye mixture and 1 mL of PVA water solution^46^ and subjecting the mixture to an ultrasonic water bath for *t*_bath_ = 10 min at 60 °C. The use of the bath allows the distribution of the size of the droplets of the emulsions to be controlled to some extent since droplets with smaller average sizes are obtained by increasing the duration of ultrasonication^46^. The *t*_bath_ value is selected to obtain an average droplet size matching the optical gain length of the used dyes, which is up to a few hundreds of μm^47^. This allows effective light amplification to be achieved by the dyes in the droplets. Multilayers of the so-prepared emulsions are realized on quartz substrates (1 × 1 cm^2^) by consecutively spin-casting the mixtures composed of PVA/SB420, PVA/LC/CM540, and PVA/LC/DCM. For the deposition of each layer, the following steps are applied: (i) casting of 0.1 mL of each mixture, (ii) realization of a film by spin-coating at 750 rpm for 1 min, and (iii) drying for 4 min under nitrogen flow.

For the fabrication of electrical contacts, 50 µL of PVA with SB420, LC/CM540, and LC/DCM is placed on a quartz substrate (2 × 2 cm^2^) coated with ITO, and a second piece of ITO-covered quartz is placed on top of the active multiphase layer. The sample is stored in ambient conditions for 1 day to let the water evaporate and the cell stabilize. The applied voltage is delivered by a DC power supply (DF1750SL3A, NDN) through the ITO electrodes.

### Optical characterization

Optical microscopy is carried out using an upright microscope (ME600 Nikon) equipped with cross polarizers. Confocal microscopy is performed using an inverted microscope equipped with a confocal laser scanning head (FV1000, Olympus), exciting samples with a laser emitting at 405 nm through a ×10 objective with a 0.4 numerical aperture. Fluorescence is collected by the same objective and measured by three photomultipliers, which simultaneously detect the blue, green, and red spectral components of the emitted light. Absorption spectra are measured by means of a Jasco V-550 system, while fluorescence spectra are measured by a spectrofluorometer (Cary Eclipse, Varian). To measure the PL temporal evolution, samples are excited by the second harmonic of a pulsed laser (Legend, Coherent, emission wavelength of 400 nm, pulse duration of 90 fs, and repetition rate of 1 kHz), and the emission is analysed by a streak camera (C10910, Hamamatsu) equipped with a spectrometer (Acton SpectraPro® SP-2300, Princeton Instruments).

The lasing properties are studied by exciting samples at 405 nm by the third harmonic of a ns pulsed Nd:YAG laser (Surelite II, Continuum) coupled to a parametric oscillator (mod. Horizon I, Continuum). The excitation beam is shaped into a stripe with a size of 0.5 × 5 mm^2^ (height × width), and the light emitted from the sample edge is collected by an optical fibre and analysed with an Ocean Optics spectrometer equipped with a diode array detector. The stability of the oWL is studied by continuously pumping the device by the third harmonic of a pulsed Nd:YAG laser (emission wavelength of 355 nm, pulse width of approximately 10 ns, and repetition rate of 10 Hz) and by measuring the emission in real time using a monochromator (iHR320, Jobin Yvon) equipped with a charge coupled device detector (Symphony, Jobin Yvon). To this aim, the oWL device is placed in a chamber, allowing for measurement of the operational lifetime both in air and vacuum (4 × 10^−2^ mbar). The divergence of the output beam of the white lasers is measured based on the spatial intensity profiles at various distances, *D*, from the light source, as schematized in Fig. S5a. A mask with a slit aperture (width of 1 mm and height of 10 mm) is positioned in front of the light sources at various distances *D*. The measurement of the intensity of the light transmitted through this mask as a function of the position along the axis perpendicular to the sample plane (*x*) allows the spatial intensity profile of the output beam and the spot size (calculated as the FWHM of the beam profile) to be determined.

## Supplementary information

Supplementary Information
