# Supplementary material for: Electrically controlled white laser emission through liquid crystal/polymer multiphases
Source: Light Sci Appl. 2020 Feb 11;9:19. doi: 10.1038/s41377-020-0252-9 (PMC7012818; doi:10.1038/s41377-020-0252-9)
Supplement: Supplementary file 1 — Supplementary Information [file 41377_2020_252_MOESM1_ESM.docx]

Supplementary Information

**Electrically controlled white laser emission through liquid crystal/polymer multiphases**

Alina Adamow^1,2^, Adam Szukalski^2^, Lech Sznitko^1^, Luana Persano^2,*^, Dario Pisignano^2,3^, Andrea Camposeo^2,*^ and Jaroslaw Mysliwiec^1,*^

*^1^Faculty of Chemistry, Wroclaw University of Science and Technology, Wybrzeze Wyspianskiego 27, 50-370 Wroclaw, Poland.*

**E-mail:* [*jaroslaw.mysliwiec@pwr.edu.pl*](mailto:jaroslaw.mysliwiec@pwr.edu.pl)

*^2^NEST, Istituto Nanoscienze-CNR and Scuola Normale Superiore, Piazza S. Silvestro 12, I-56127 Pisa, Italy.*

**E-mail:* [*luana.persano@nano.cnr.it*](mailto:luana.persano@nano.cnr.it)*,* [*andrea.camposeo@nano.cnr.it*](mailto:andrea.camposeo@nano.cnr.it)

*^3^Dipartimento di Fisica, Università di Pisa, Largo B. Pontecorvo 3, I-56127 Pisa, Italy*

#
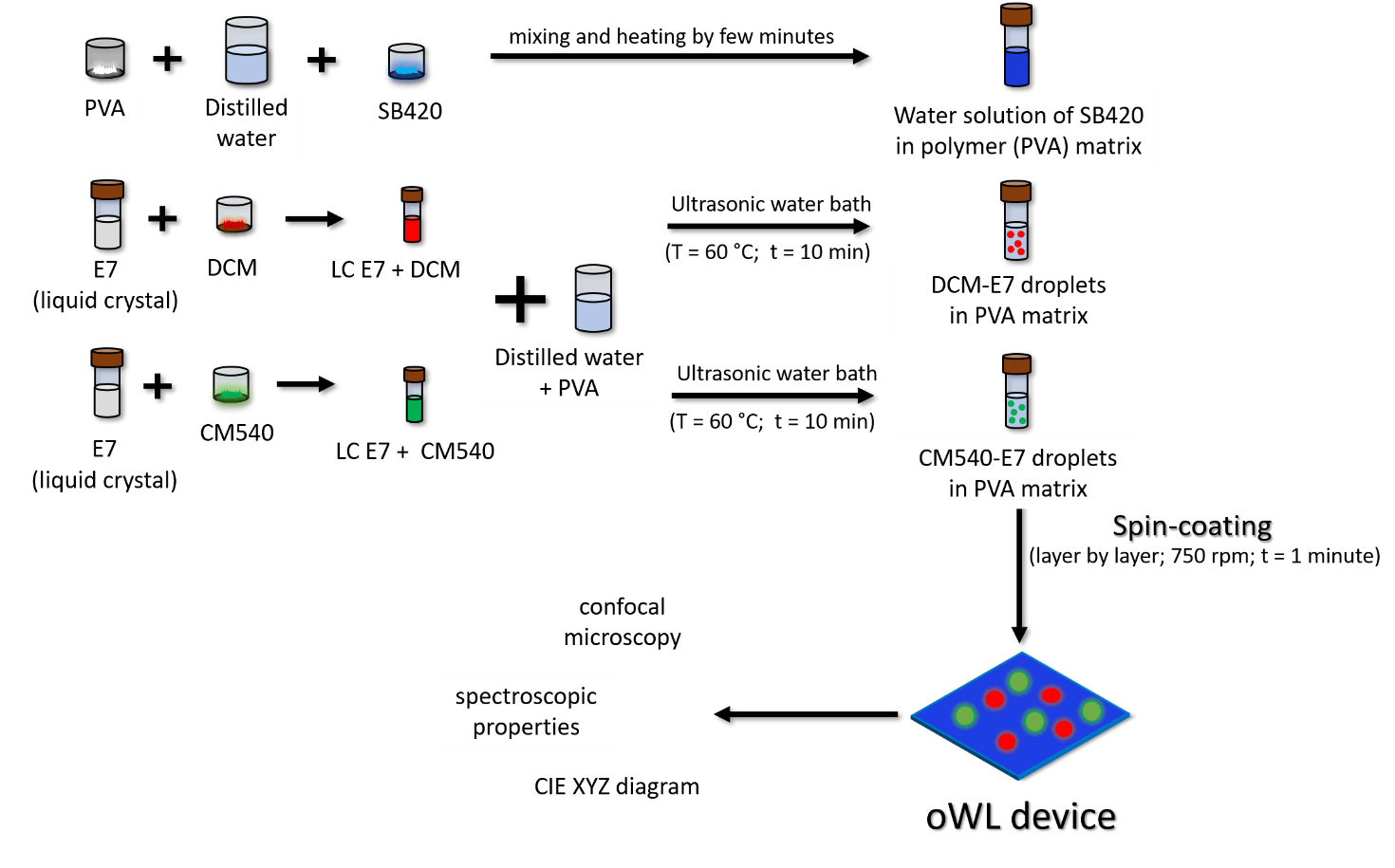


# Figure S1. Schematic representation of the steps for the organic white laser (oWL) preparation.

#
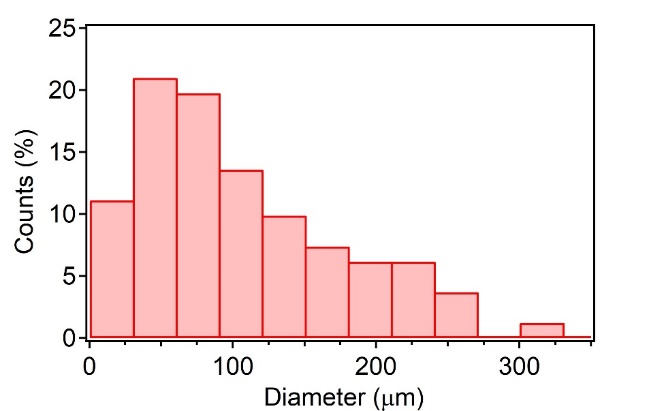


# Figure S2. Distribution of the size of the LC droplets. Sonication time, t_bath_= 10 min.

#

# Figure S3. (a),(b) Confocal fluorescence micrographs of PVA with LC/CM540 (a), and PVA with SB420 and LC/DCM (b). (c),(d) Corresponding lasing spectra, obtained with different excitation fluences of samples shown in (a) and (b), respectively. Excitation fluence values (in mJ cm^-2^) are shown in each panel and correspond to the shown spectra (from bottom to top). (e),(f) Corresponding plots of the emitted intensity *vs*. the excitation fluences. The dashed and continuous lines are linear fits to the data in the range of excitation below and above threshold, respectively. Measured threshold values are given at the top of each plot.

#
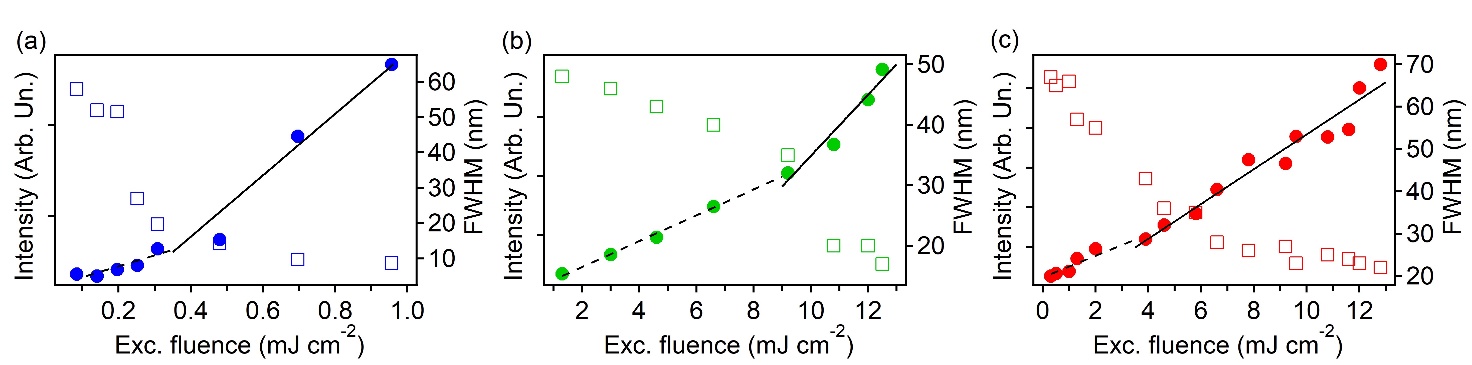


# Figure S4. Dependence of the intensity (full symbols, left vertical scale) and FWHM (open symbols, right vertical scale) of the emission spectra on the excitation fluence, measured for samples containing the single dyes: SB420 (a), CM540 (b) and DCM (c). The dashed and continuous lines are linear fits to the data in the range of excitation below and above threshold, respectively.

# Figure S5. (a) Schematic illustration of the set-up used for the measurement of the divergence of the output beam of the oWLs. (b) Intensity spatial profile of the output beam measured at *D*=30 mm (triangles) and *D*=63 mm (circles) from the light source. The dashed lines are fits to the data with a Gaussian curve. Data measured at *D*=30 mm and *D*=63 mm are normalized to their maximum value, respectively. Excitation fluence: 15 mJ cm^-2^.

#
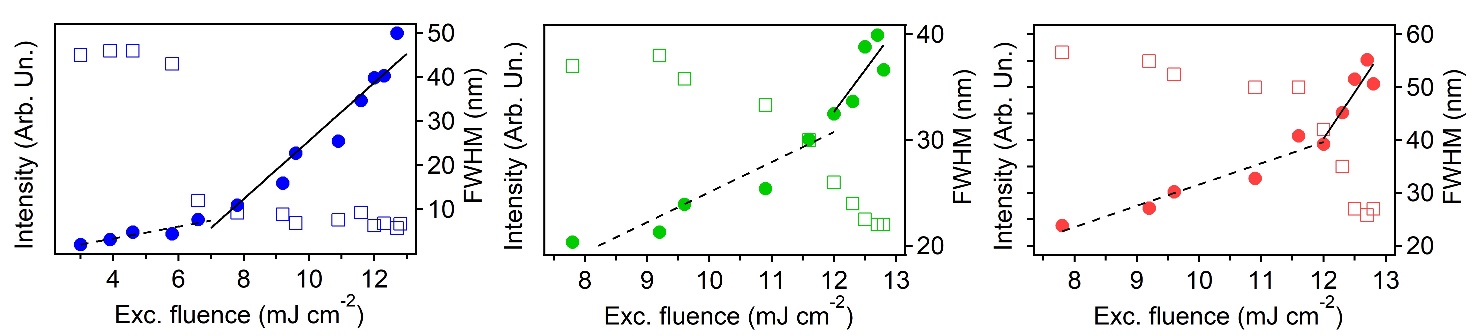


# Figure S6. Dependence of the oWL intensity (full symbols, left vertical scale) and FWHM (open symbols, right vertical scale) on the excitation fluence. Data are obtained by the spectra shown in Figure 2h of the main text. Intensities are measured by integrating the emission in a spectral interval corresponding to the emission peak of SB420 (a), CM540 (b) and DCM (c). Used spectral ranges are: 435-450 nm (a), 526-536 nm (b), and 600-610 nm (c), respectively. The dashed and continuous lines are linear fits to the data in the range of excitation below and above threshold, respectively.

#
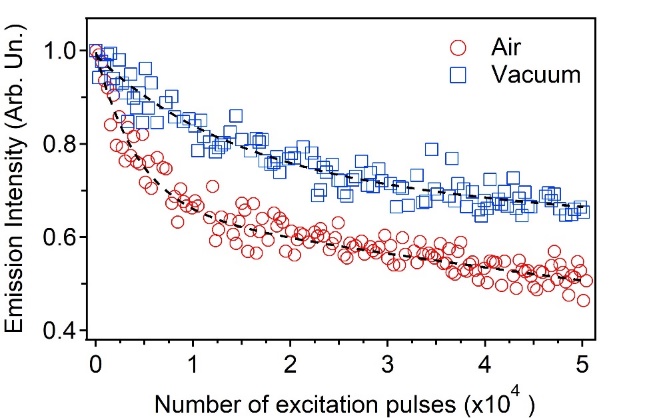


# Figure S7. Emission intensity upon increasing the number of the excitation pulses. Data are collected in air (red circles) and under vacuum conditions (blue squares). Black dashed lines are guide for the eyes. Excitation fluence: 13 mJ cm^-2^.

**Figure S8.** Dependence of the CIE chromaticity coordinates of the oWL emission, upon varying the excitation fluence.

# Figure S9. CIE coordinates of an oWL device upon applying consecutively 0 V (blue circle), 5 V (red circle) and 0 V (green circle).

#
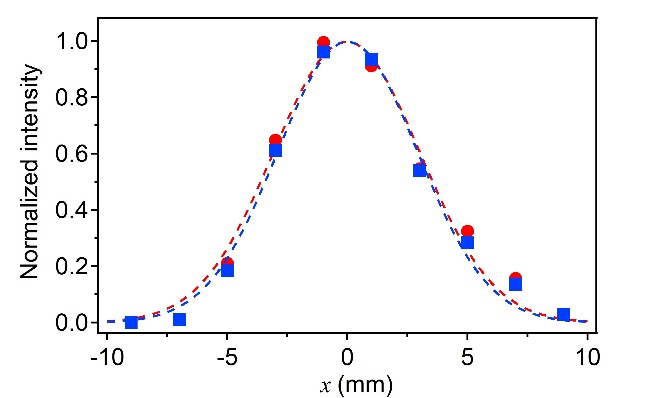


# Figure S10. Comparison of the spatial intensity profile of the output beam of the white emitting laser devices with an applied voltage of 0 V (red circles) and 5 V (blue squares). Red and blue dashed lines are fits to the data acquired at 0 V and 5 V, respectively, by a Gaussian profile. Data acquired at 0 V and 5 V are normalized to their maximum value, respectively. Excitation fluence: 15 mJ cm^-2^.

# Table S1. Photoluminescence lifetimes of SB420, CM540 and DCM chromophores measured in an oWL and in samples containing individual dyes.

| Sample | PL lifetime in individual samples [ns] | PL lifetime in oWL [ns] |
| --- | --- | --- |
| SB420 | 0.7 | 0.8 |
| CM540 | 2.7 | 2.0 |
| DCM | 1.6 | 2.4 |
